# Intercellular Transfer of Oncogenic H-Ras at the Immunological Synapse

**DOI:** 10.1371/journal.pone.0001204

**Published:** 2007-11-21

**Authors:** Oded Rechavi, Itamar Goldstein, Helly Vernitsky, Barak Rotblat, Yoel Kloog

**Affiliations:** 1 Department of Neurobiochemistry, The George S. Wise Faculty of Life Sciences, Tel-Aviv University, Tel-Aviv, Israel; 2 Immunology Program, Cancer Research Center, Chaim Sheba Medical Center, Tel Hashomer and Sackler School of Medicine, Tel-Aviv University, Tel-Aviv, Israel; University Paris Sud, France

## Abstract

Immune cells establish dynamic adhesive cell–cell interactions at a specific contact region, termed the immunological synapse (IS). Intriguing features of the IS are the formation of regions of plasma membrane fusion and the intercellular exchange of membrane fragments between the conjugated cells. It is not known whether upon IS formation, intact intracellular proteins can transfer from target cells to lymphocytes to allow the transmission of signals across cell boundaries. Here we show by both FACS and confocal microscopy that human lymphocytes acquire from the cells they scan the inner-membrane protein H-Ras, a G-protein vital for common lymphocyte functions and a prominent participant in human cancer. The transfer was cell contact-dependent and occurred in the context of cell-conjugate formation. Moreover, the acquisition of oncogenic H-RasG12V by natural killer (NK) and T lymphocytes had important biological functions in the adopting lymphocytes: the transferred H-RasG12V induced ERK phosphorylation, increased interferon-γ and tumor necrosis factor-α secretion, enhanced lymphocyte proliferation, and augmented NK-mediated target cell killing. Our findings reveal a novel mode of cell-to-cell communication—allowing lymphocytes to extend the confines of their own proteome—which may moreover play an important role in natural tumor immunity.

## Introduction

Ras proteins play a key role in the determination of cell fate [1] and malignant transformation [2]. They are also prominent participants in immune-cell function [3], [4], and recent studies indicate a link among impaired Ras signaling and T cell anergy. Signals of the active (GTP-bound) Ras from the inner plasma membrane (PM) and from endomembranes activate a multitude of effectors including the Raf/MEK/ERK cascade, phosphatidylinositol-3-OH kinases, and Ral-guanine nucleotide exchange factors [5]. Ras proteins are highly mobile, redistribute rapidly between the PM and endomembranes [6]–[8], and even diffuse out of cells when the PM is pharmacologically perforated [9]. Interestingly, during the formation of an immunological synapse (IS) both extracellular receptors [10] and membrane patches are exchanged between cell-conjugates; a process also termed trogocytosis [11], [12]. Although Ras resides exclusively inside the cell and is not a transmembrane protein, it is conceivable that this highly mobile signal-transducing molecule can be transferred to lymphocytes from the cells they scan. Importantly, no studies have shown that intracellular, inner-membrane proteins can be exchanged between immune cells. We hypothesized that the IS may permit the intercellular transfer of inner-membrane associated proteins—such as Ras—during conjugation from target cells into lymphocytes.

## Results and Discussion

To test our hypothesis we used the human class I-deficient B cell line 721.221 (B721). This antigen presenting cell (APC) readily forms IS with natural killer (NK) and T cells. We generated B721 lines stably expressing green fluorescent protein (B721−GFP, control cells) or the GFP-tagged constitutively active H-Ras mutant G12V (B721−GFP-H-RasG12V). Because B721 is highly susceptible to NK cell-mediated killing, we first tested H-RasG12V transfer in the context of the cytolytic IS. Thus, freshly isolated human CD56+ NK cells from the peripheral blood of healthy donors (*n*>10) were co-cultured with the B721−GFP or B721−GFP-H-RasG12V target cells for various periods of time. The CD56+ NK cells were then labeled with red fluorophore-tagged CD56 monoclonal antibodies (mAbs), vigorously resuspended in 5 mM EDTA/phosphate-buffered saline (PBS) to disrupt the CD56+NK/B721 conjugates, and analyzed by fluorescence activated cell sorter (FACS).

**Figure 1:**
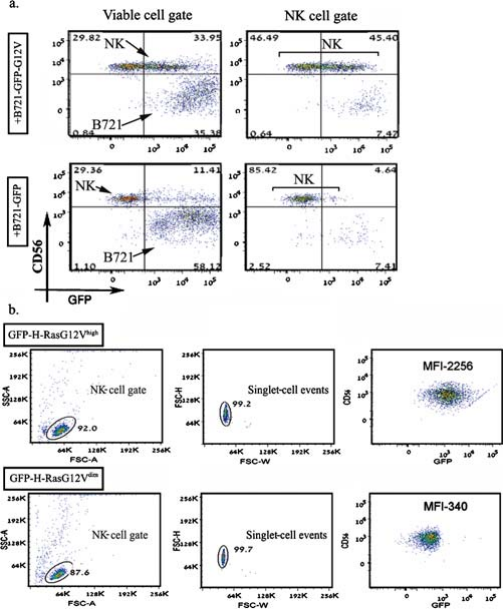
GFP-H-RasG12V but not GFP is transferred from B721 cells into NK cells. (a) Freshly isolated CD56+ peripheral blood lymphocytes (PBLs) from healthy subjects were co-cultured with B721−GFP cells (lower panel) or B721−GFP-G12V cells (upper panel) for 2 h. To disrupt cell conjugates, the collected cells were treated with 5 mM EDTA/Ca^2+ ^-free PBS on ice for 30 min, vortexed, stained with allophycocyanin-conjugated anti-CD56 mAbs, and then analyzed by flow cytometry (FACSAria™). Dot plots on the left depict the total live-cell gate, and plots on the right show gating of mostly NK cells, as determined by FSC and SSC. Values represent the relative percentages of events occurring within each quadrant. Results shown are from a typical experiment out of >10 performed in duplicates (data collected from ∼10 000 single cell events). (b) Co-cultures of CD56+ PBL and B721−GFP-G12V cells were sorted by FACSAria™ into GFP-labeled (GFP^high^; upper panel) and unlabeled (GFP^negative^; lower panel) NK cells. Left and middle plots show the FSC/SSC gating strategy used to obtain these two NK cell populations, each composed of >99% singlet-cell events. Values in the right plots represent the MFI of the green filter. Data are from a typical experiment out of >5 performed.

We found that a large proportion of the NK cells in co-culture with B721-GFP-H-RasG12V acquired GFP tagged H-RasG12V molecules from their co-culture partners (Fig. 1a, right upper dot plot−right upper quadrant; 45.4% CD56^+ ^GFP^+/high^ events) compared to the NK cells co-cultured with B721-GFP cells (right lower dot plot−right upper quadrant; 4.6% CD56^+^ GFP^+^ events). As seen in the viable cell-gate containing both NK and B721 cells and their conjugates (Fig. 1a, left hand dot plots), the expression of either GFP-H-RasG12V or GFP molecules in the respective B721-transfectants was similar (right lower quadrant within left hand plots; CD56^−^ GFP^high^ events). The acquisition of GFP-H-RasG12V was rather rapid, evident by 15 min and maximal after 2–3 hours (Supporting Text S1, Fig S1). Interestingly, the mean fluorescence intensity (MFI) of the GFP-H-RasG12V molecules adopted by some of the NK cells was almost as high as the MFI of the B721−GFP-H-RasG12V cells, indicating that a significant proportion of the H-RasG12V molecules were transferred from targets to lymphocytes. To further confirm that the GFP-H-RasG12V molecules had been acquired by NK singlet (non-conjugated) cells, we used a digital cell sorter and a stringent multi-parameter gating strategy to physically sort out from the NK/B721-GFP-H-RasG12V co-cultures two populations of GFP-H-RasG12V^high^ and GFP-H-Ras12V^negative^ NK singlet cells. We thus obtained two populations of highly purified (>99%) singlet NK cells with significantly different GFP-H-RasG12V contents (Fig. 1b) confirming that GFP-H-RasG12V was indeed transferred into the NK cells.

**Figure 2:**
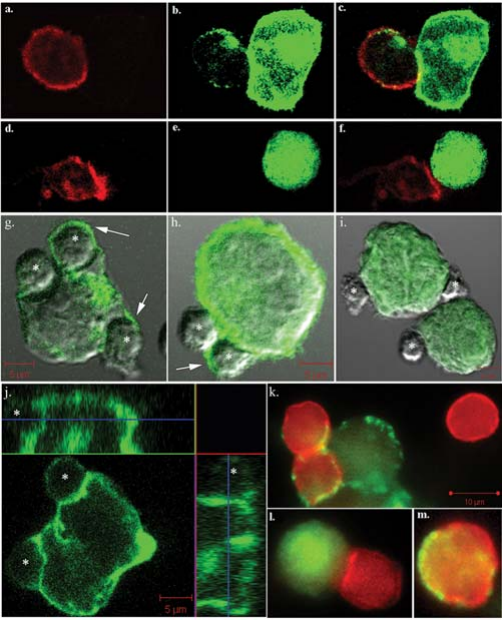
Transfer of GFP-H-RasG12V from B721 cells to immune cells during conjugate formation. (a–f) CD4+HVS cells were co-cultured, for 2–3 h with B721−GFP-H-RasG12V (a–c) or B721−GFP (d–f). The cells were collected and cytospun onto slides, fixed, stained with anti-CD45-phycoerythrin-Cy5 (red) mAbs, and then subjected to dual-image confocal microscopy (red for T cells; green for GFP). (g–i) Overlay of Nomarski and confocal green fluorescence of freshly isolated NK cells (* denotes smaller cells) co-cultured with B721−GFP-H-RasG12V (g, h) or with B721−GFP (i) larger green cells. (j) Green fluorescence image of a B721−GFP-G12V cell conjugated with two NK cells. The two insets depict the reconstituted Z scans. (k–m) Merged epifluorescence microscopy of red NK cells (CD45 phycoerythrin-Cy5+) and green B721 cells, co-cultured and processed as above. Panel k depicts B721−GFP-H-RasG12V conjugated with two NK cells that captured GFP-H-RasG12V and a non-conjugated NK cell that did not take up GFP. In panel l, in contrast, the NK cell conjugated with B721−GFP did not acquire any GFP. Panel m represents a sorted GFP-H-RasG12V^high^ singlet-NK cell (as described in Fig. 1b) cytospun onto a slide. Arrows indicate GFP-H-RasG12V captured by the NK cells. Data are from a typical experiment out of >5 performed.

Next we used indirect immunofluorescence staining with anti-Ras mAbs to determine whether the transferred H-Ras was localized within the cells or attached to the cell surface. Intracellular staining, as analyzed by FACS was detected only in fixed and permeabilized GFP-H-RasG12V^high^ NK cells (Fig. S2d). Without PM permeabilization no H-Ras staining was detected in the same cell population (Fig. S2f). Confocal and epifluorescence microscopy of conjugates of NK/B721−GFP-H-RasG12V (Fig. 2a–c, g, h, j; confocal, Fig. 2k; epifluorescence) and of NK/B721−GFP (Fig. 2d–f, i; confocal, Fig. 2l; epifluorescence), as well as of sorted GFP-H-RasG12V^high^ NK cells (Fig. 2m, epifluorescence), showed that GFP-H-RasG12V was integrated into the PM of the NK cells. It thus seems that upon formation of cell conjugates, GFP-H-RasG12V simply ignored cell boundaries and was distributed in significant amounts in the PM of both the ‘acceptor’ and ‘donor’ cells. The anti-Ras mAbs staining experiments, furthermore, suggest that the transferred GFP-H-RasG12V was incorporated into the adopting lymphocyte's membrane in the correct orientation, i.e. into the inner cell membrane leaflet. In agreement with these data, McCann et al have recently demonstrated that cell surface receptors transferred from B721 to NK cells also assume a normal in/out transmembrane orientation in the adopting lymphocyte [13].

**Figure 3:**
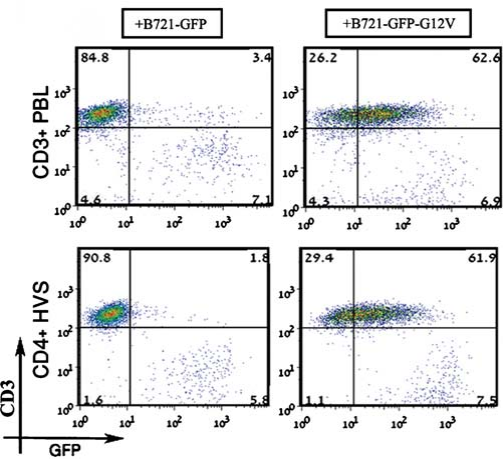
GFP-H-RasG12V is also transferred from B721 cells into T cells. Fresh CD3+ PBLs isolated from healthy blood donors (upper panel) or CD4+HVS cells (lower panel) were co-cultured with B721−GFP cells (left panel) or B721−GFP-G12V (right panel) for 3 hours. Cells were collected and treated as described in figure 1a, stained with anti-CD3-allophycocyanin, and then analyzed by FACSCalibur™. Results represent a typical experiment out of >10 independent experiments (data from ∼10 000 single cell events).

It was of interest to also study whether this novel mode of GFP-H-RasG12V transfer occurs during the formation of a non-cytolytic IS. We therefore used freshly isolated human NK-depleted CD3+ T cells purified from the peripheral blood of healthy donors (*n*>10). The T cells were co-cultured for up to 4 h with the B721 transfectants, and the co-cultures were then stained with red fluorescence-tagged anti-CD3 mAbs and analyzed by FACS. We found that the peripheral blood-derived CD3+ T cells (Fig. 3), like the NK cells (Fig. 1a), efficiently acquired GFP-H-RasG12V from B721−GFP-H-RasG12V cells, even though these T cells were B721-naïve and did not induce target-cell killing (not shown). In comparison no noteworthy uptake of GFP molecules from the B721−GFP control cells was observed (Fig. 3). Acquisition of GFP-H-RasG12V was increased when the T cells were activated with anti-CD3 mAbs (not shown). Moreover, herpes Saimiri-transformed human CD4+ T cells (CD4+HVS, Fig. 3) that do not express perforin and non-cytotoxic Jurkat T cells (Fig. S3) also acquired GFP-H-RasG12V from B721−GFP-H-RasG12V cells. Overall, the FACS-based experiments along with the fluorescence confocal microscopy studies substantiated our hypothesis that GFP-H-RasG12V can be transferred from APCs into NK and T cells upon conjugate formation.

To determine whether the ability to acquire GFP-H-RasG12V from the B721 cells is restricted mainly to primary T or NK lymphocytes we examined NK92 cells, the monocytic leukemia cell lines K562 and KG1, primary CD19+ B cells and Epstein-Barr virus (EBV)-transformed lymphoblastoid cell lines (LCLs). NK92 cells readily acquired GFP-H-RasG12V from B721−GFP-G12V cells (Fig. S3). Primary B cells and LCLs also acquired GFP-H-RasG12V, albeit to a lesser extent than the NK or T cells, whereas the non-lymphocytic cell lines K562 and KG1 did not acquire GFP-H-RasG12V (Fig. S4). It thus appears that acquisition of GFP-H-RasG12V from APCs is mainly a lymphocytic characteristic.

To examine whether the ability to transfer H-RasG12V is limited to B721 cells we used COS7 and HEK293 cells transfected with GFP-H-RasG12V or with GFP and incubated the transfectants with CD3+ T lymphocytes. We found that CD3+ T lymphocytes readily acquired GFP-H-RasG12V but not GFP from the COS7 and HEK293 transfectants (Figs. S5). Furthermore, to determine whether the transfer of H-Ras is GFP fusion protein dependent we transfected HEK293 cells with HA (hemagglutinin) tagged H-RasG12V construct. The transfectants were then co-cultured with NK cells for 2 hours. Using intracellular staining and FACS analysis we found that HA-H-RasG12V was transferred to NK cells (Fig. S6). Thus, intercellular transfer of oncogenic H-Ras is independent of a specific fusion protein and H-Ras can be acquired from various cell types including non-classical APCs.

**Figure 4:**
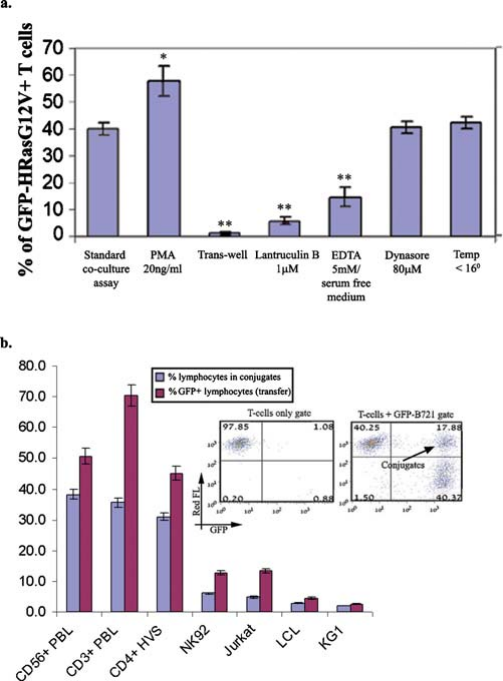
Transfer of GFP-H-RasG12V from B721 into lymphocytes is dependent on cell contact and the actin cytoskeleton. (a) CD3+T cells were co-cultured with B721−GFP-H-RasG12V cells for 3 h. The co-cultures were treated as indicated or left untreated. In the trans-well experiments, T cells were separated from B721 cells by a 0.4-µm-pore membrane. Acquisition of GFP-H-RasG12V by T cells under the different experimental conditions was then determined by FACS, as described in Methods. (b) The different cell types were co-cultured with B721−GFP-H-RasG12V for 3 h, and GFP acquisition by T cells was determined as above. The same cell types were co-cultured for 45 min with B721-transfectants at a ratio of 1∶1, and the percentage of cell conjugates was then determined by FACS analysis (double-positive cells, as shown in inset). Experiments were performed in duplicates and repeated >3 times. Data was from ∼10,000 single cell events and the FlowJo V6.4.7 population comparison platform (probability binning Chi(T) algorithm) was used to determine the statistical significance of the difference observed between the indicated treatments and control group. Bars represent mean±SD. *P<0.01, ** *P*<0.001.

We next examined whether close contact between T or NK lymphocytes and B721 cells is obligatory for transfer of GFP-H-RasG12V to lymphocytes. We found that physical separation of NK or T lymphocytes from the B721-GFP-H-RasG12V cells by means of a semi-permeable transwell membrane (0.4-µm pore size) completely blocked the transfer of GFP-H-RasG12V (Fig. 4a). Moreover, in examining whether the ability of various cell types to form conjugates with B721 transfectants correlates with their ability to acquire GFP-H-RasG12V, we observed a strong positive correlation between the extent of their GFP-H-RasG12V acquisition and the percentage of cell conjugates formed (Fig. 4b). In addition, the actin cytoskeleton inhibitor latrunculin B known to inhibit the formation of a ‘mature’ IS as well as trogocytosis between T cells and their targets [14] abolished both the intercellular transfer of GFP-H-RasG12V (Fig. 4a) and conjugate formation (Fig. S7). The intercellular transfer of GFP-H-RasG12V was also reduced in the presence of 5 mM EDTA under serum-free conditions a treatment that also interfered with cell conjugate formation (Fig. 4a and Fig. S7). Conversely, transfer of GFP-H-RasG12V was increased by the addition of phorbol 12-myristate 13-acetate (PMA) that also increased conjugate formation (Fig. 4a and Fig S7). The transfer, moreover, took place at temperatures <16°C and was unaffected by dynasore that is a potent dynamin inhibitor; treatments known to interfere with clathrin-mediated endocytosis [15] (Fig. 4a). Taken together these experiments indicate that the uptake of shed membrane vesicles, i.e. exosomes [16], [17], or endocytosis of shed H-Ras proteins are not essential for this mode of intercellular communication. The intercellular transfer of GFP-H-RasG12V from B721 cells to T and NK lymphocytes, nonetheless, required cell conjugation, IS formation, and possibly the subsequent membrane-transfer at the IS.

Using HEK293 or COS7 transfectants we found that GFP-H-Ras wild type (wt), like GFP-H-RasG12V, was also transferred from ‘donor’ cells to lymphocytes, indicating that the transfer process is independent of the GDP/GTP binding state of Ras (Figs. S8). Also, the fusion product of the double palmitoylated and farnesylated C-terminal tail of H-Ras and GFP (tH) [18] was efficiently transferred into lymphocytes (Fig. S8) indicating that the lipid-modified tail of H-Ras is sufficient to allow this process to occur.

We next examined the relationship between the observed Ras transfer and trogocytosis. We used the cell surface receptor CD86 as a marker for trogocytosis as previously described [10], [11]. Thus, NK cells that express low levels of CD86 were co-cultured for 2 hours with either B721-GFP or B721-GFP-H-RasG12V cells, both expressing high levels of CD86. Subsequently, we analyzed by FACS the NK cells for the acquisition of both GFP-H-RasG12V and CD86. As Shown in Fig. S9a, we observed a strong correlation (r^2^ = 0.63; *P*<0.001) between GFP-H-RasG12V acquisition and trogocytosis as measured by the uptake of CD86. These results suggest that the two processes are linked. Notably, acquisition of activated Ras did not appear to increase trogocytosis per se, as the acquisition of CD86 was comparable in NK cell that were co-cultured with B721-GFP-H-RasG12V or with B721-GFP (Fig. S9b).

**Figure 5:**
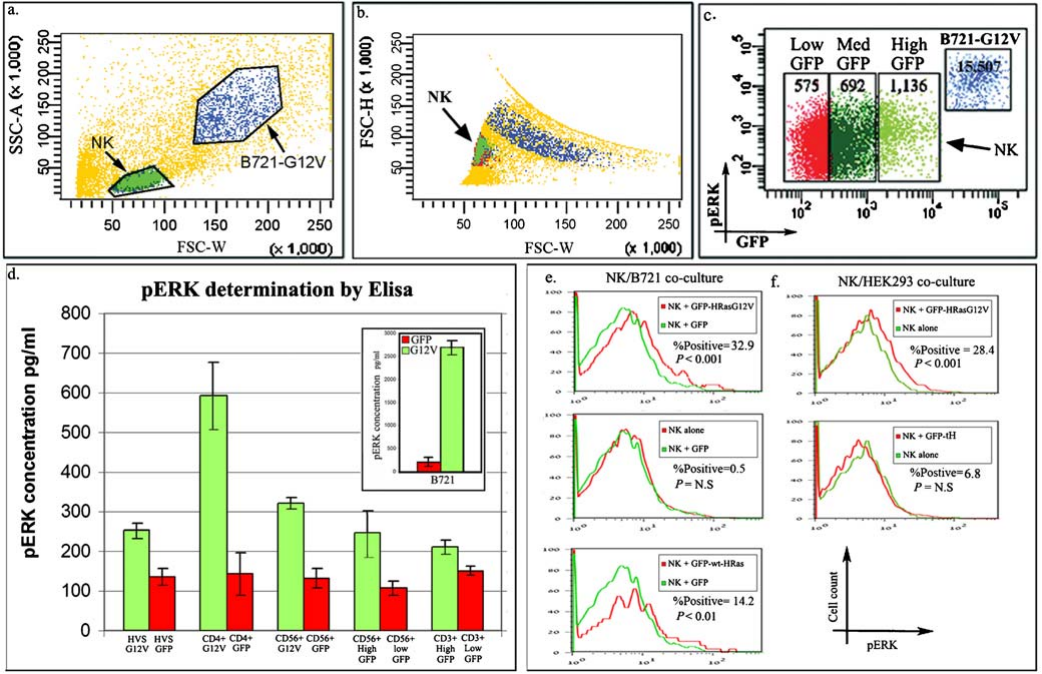
The acquisition of oncogenic H-RAS at the IS results in ERK phosphorylation within the acquiring lymphocytes. (a–c) NK cells were co-cultured with B721−GFP-H-RasG12V for 2 hours. The collected cells were fixed/permeabilized, stained for intracellular pERK and analyzed by FACS. Dot plots a+b depict the careful gating strategy used to distinguish singlet NK cells (green) from duplets and B721 cells (blue), and dot plot c shows GFP-H-RasG12V vs. pERK levels. The NK population was divided into three subpopulations (red, dark green and bright green) according to their GFP content. Bold numbers indicate the MFI of pERK. Shown is a representative experiment out of the five performed (data from ∼20,000 events). (d) T and NK lymphocytes from various co-cultures of B721-GFP-H-RasG12V (G12V) or B721-GFP (GFP) were either isolated using the MACS^® ^cell separation system or sorted (as described in Fig. 1b) into GFP-H-RasG12V^high^ and GFP-H-RasG12V^low^ cells. The cells were then lysed and pERK levels were determined by ELISA. Total pERK levels in lysates of B721-H-RasG12V relative to B721-GFP were also determined in parallel as controls for test performance (inset). Assays were performed in duplicates and bars represent the mean±SD from a typical experiment out of >5 performed. (e) NK cells were co-cultured with B721 cells stably expressing either GFP-H-RasG12V, GFP-H-Ras(wt) or GFP for 2 hours. The upper overlay histogram depicts total pERK levels within GFP-H-RasG12V^high^ NK cells compared to control NK cells. The middle overlay histogram compares total p-ERK levels in NK cells co-cultured with B721-GFP versus control NK cells. The lower overlay histogram shows p-ERK levels within GFP-H-Ras(wt)+ NK cells compared to control NK cells. (f) NK cells were co-cultured with HEK293 transfected with either GFP-H-RasG12V or GFP-tH constructs for 2 hours. The overlay histograms depict total p-ERK levels within GFP^high^ NK cells in the two co-cultures (red lines) compared to control NK cells (green lines). The FlowJo V6.4.7 population comparison platform was used to calculate %Positive cells found in the sample compared to control (super enhanced D-Max algorithm), and the probability binning Chi(T) algorithm was used to determine the statistical significance of the difference between samples (*P* value).

To test the functional consequences of the transferred H-RasG12V in the adopting lymphocyte, we recorded phospho-ERK (pERK) levels as readout of the activation of the Ras/Raf/MEK/ERK cascade in NK cells that were co-cultured with B721−GFP-H-RasG12V. Thus, freshly isolated human CD56+ NK cells from the peripheral blood of healthy donors were co-cultured with the B721−GFP-H-RasG12V target cells for two hours. By intracellular immunofluorescence staining with anti-pERK mAbs, followed by FACS analysis (Fig. 5a–c), we demonstrate an increase in the intracellular levels of pERK within the NK cells that acquired H-RasG12V. This increase was dose-dependent, i.e., the more GFP tagged H-RasG12V molecules acquired from the co-culture partners, the higher the intracellular pERK levels detected (Fig. 5c; numbers in bold). Additionally, two hours after co-culturing we sorted out, using a digital cell sorter, from the NK (or T) cells and B721-GFP-H-RasG12V co-cultures two separate populations of GFP-H-RasG12V^high^ and GFP-H-RasG12V^negative^ NK (or T) singlet cells. Subsequently, we analyzed the levels of pERK in the cell-lysates obtained from these two different populations of sorted NK (or T) cells by ELISA kits (Fig. 5d). As a positive control, we analyzed in parallel the pERK levels in the lysates of the B721-GFP-H-RasG12V transfectants (figure inset). We found that pERK levels were significantly increased in the NK or T cells that acquired GFP-H-RasG12V, when compared to equivalent NK or T cells purified from the same co-cultures that, in contrast, did not acquire GFP-H-RasG12V. In addition, FACS analysis (Fig. 5e) indicated that NK cells that acquired GFP-H-RasG12V exhibit a higher increase in the intracellular levels of pERK compared with NK cells that acquired GFP-H-Ras(wt) or NK cells that were co-cultured with B721-GFP. Similarly, NK cells that were co-cultured with HEK293 expressing GFP-H-RasG12V displayed higher levels of pERK compared with NK cells co-cultured with HEK293 expressing the inactive GFP-tH (Fig. 5f). Thus the acquired H-Ras activity appears to correlate with increased ERK phosphorylation independent of the donor cell line type.

**Figure 6:**
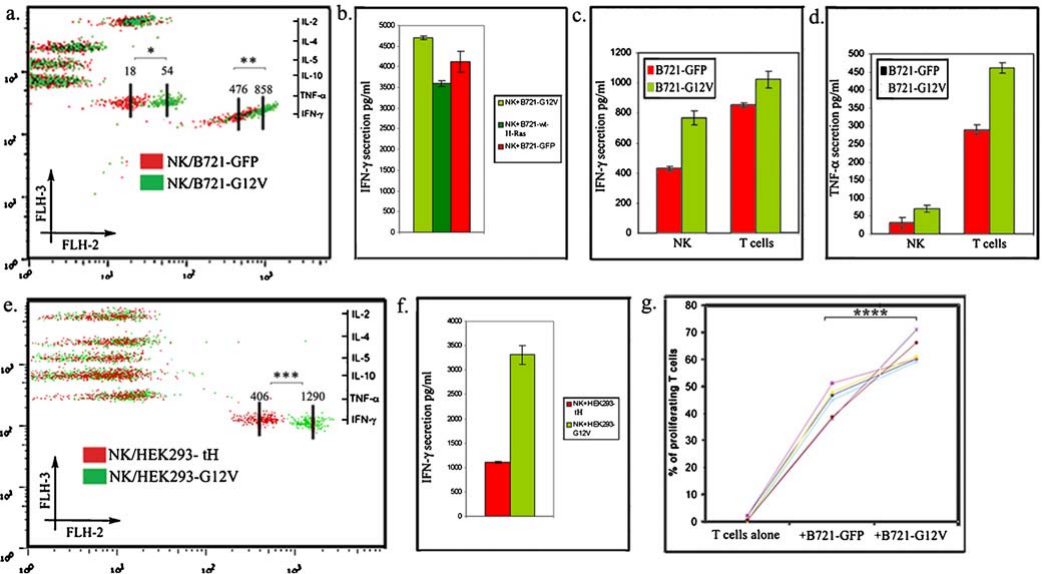
Functional consequences of H-RasG12V acquisition. Purified CD56+ NK or CD3+ T cells were co-cultured for 24 hours with either B721 or HEK293 transfectants as indicated. The supernatants were collected and levels of IL-2, IL-4, IL-5, IL-10, IFN-γ and TNF-α were determined using the Th1/Th2 CBA kit (∼300 beads collected per analyte). (a) Overlay of the two dot plots obtained from supernatants collected from either NK/B721-GFP or NK/B721-G12V co-cultures. Numbers represent the MFI of the respective cytokine. (b) The raw data was computed with the BD™ CBA Software to calculate the levels (pg/ml) of IFN- γ in the indicated supernatants. (c–d) NK and T cells were compared for cytokine IFN- γ (c) or TNF-α (d) secretion in response to either B721-GFP or B721-G12V. (e) Overlay of the two dot plots from supernatants collected from either NK/HEK293-tH or NK/HEK293-G12V co-cultures. (f) The raw data was computed with the BD™ CBA Software to calculate the levels (pg/ml) of IFN- γ in the indicated supernatants. Bars represent the mean±SD of triplicates from a typical experiment out >5 performed. (g) Freshly isolated PBLs were labeled with CFSE and co-cultured with irradiated B721−GFP or B721−H-RasG12V (modified MLR). After 7 days, viable T lymphocytes in the co-cultures were analyzed by FACS for CFSE-dilution (% proliferation). Dots linked by lines represent results from the same donor. *, *P* = 0.001; **, *P* = 0.01; ***, *P*<0.001 (n = 300), and ****P = 0.02 (n = 6).

Because Ras-induced activation of ERK has been shown to increase cytokine secretion and cell proliferation [19], [20] we examined whether these effector functions were indeed enhanced in the lymphocytes that acquired GFP-H-RasG12V. As shown in figures 6a–f, IFN-γ and TNF-α secretion–as assayed by the Th1/Th2 cytometric bead array method–was augmented by the acquisition of GFP-H-RasG12V regardless of whether oncogenic H-Ras was transferred from B721 (Fig. 6a–d) or HEK293 (Fig. 6e and f) cells. An increase in IFN- γ secretion by NK cells was recorded when the cells acquired H-RasG12V but not when they acquired H-Ras (Fig. 6b). Notably, different pools of B721 transfectants were used in the experiments depicted in figures 6a–d indicating that the increase was independent of any inherent quality of a particular B721 pool; this incremental increase was especially impressive considering that B721 are by themselves potent stimulators of NK and T cells. Importantly, in analogous experiments performed with HEK293 (a non APC line) we found that NK cells co-cultured with GFP-H-RasG12V transfectants secreted higher amounts of IFN-γ compared to NK cells co-cultured with the GFP-tH transfectants (Fig. 6e and f). In addition, in the context of the mixed lymphocyte reaction, the proliferation of CD3+ T cells (Fig. 6g), as measured by vital dye dilution, was significantly enhanced in the peripheral blood lymphocyte (PBL) samples co-cultured with B721-GFP-H-RasG12V compared to identical PBL samples co-cultured with B721-GFP cells. Thus, these findings support the notion that GFP-H-RasG12V transfers from B721 to T and NK cells in a mode that permits signal transduction with ensuing biologically relevant effects in the adopting cell.

**Figure 7:**
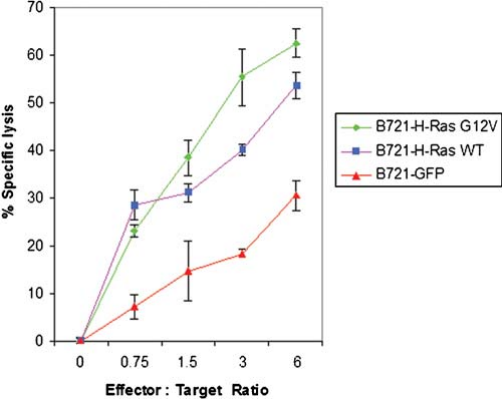
Enhancement of killing by human NK cells after acquisition of H-RasG12V. NK cells were isolated from the peripheral blood of four independent donors. Next, the various B721 transfectants were distributed into U-bottom 96-well plates to which the NK cells were added to obtain different effector to target ratios. The percentage of cell lysis was measures by flow cytometry by computing the relative number of viable (unlysed) GFP+ target cells, as determined by propidium iodide exclusion (see Materials and Methods for further details). Assays were performed in triplicates and data points represent mean±SD (error bars). This result is representative of four independent experiments.

It is well established that ERK2 is required for NK effector function and regulates the mobilization and redistribution of the cytolytic granules towards the synaptic region [21]. Recently it was shown that NK cell cytotoxicity is augmented by sustained activation of the MEK1/2–ERK pathway, but is significantly reduced by either pharmacologically blocking ERK activation (phosphorylation) or knockdown of ERK2 by RNAi [22]. Given our findings that the transferred GFP-H-RasG12V molecules signal in the adopting NK cells to increase ERK-phosphorylation and the prominent role that pERK plays in NK effector functions, it was of interest to investigate whether the acquisition of H-RasG12V indeed enhances NK cell mediated cytotoxicity. Polyclonal human NK cells freshly isolated from PBL and stimulated for 24 h with IL-2, kill very efficiently B721 cells, as determined by a FACS-based fluorolysis assay. However this cytotoxic activity becomes moderate when effector to target ratio is low (below 5) and the co-culture duration is reduced to 90 minutes (data not shown). Thus, we investigated whether under these particular experimental conditions the cytotoxicity of the polyclonal human NK cells was affected by the acquisition of H-RasG12V. We found that the cytotoxic activity of NK cells towards B721 cells was strongly enhanced upon acquisition of H-RasG12V and enhanced to a lesser extent by the acquisition of H-Ras when compared to the background cytotoxicity (Fig. 7). Interestingly, and in agreement with our results, past experiments have demonstrated an enhanced cytotoxic activity of activated NK cells towards fibroblasts transfected with oncogenic H-Ras, but not fibroblasts transfected with a different oncogene, c-Myc [23]. It can be envisioned that c-Myc which mainly localizes to the nucleus—as opposed to the membrane associated H-Ras—is less likely to be transferred at the IS and thus augment NK effector function.

The classical model of cellular immune surveillance entails transfer of intercellular information through cell-surface interactions between receptors and their ligands [24]–[26]. The present finding that lymphocytes acquire intact H-Ras from the cells they survey demonstrates a hitherto unknown immune surveillance mechanism in which lymphocytes can directly ‘sense’ and respond to changes in the intracellular proteome of the cells they scan. In this context, the transfer of membrane patches from tumor cells to lymphocytes has been documented [27], and recently trogocytosis of the ‘inhibitory’ HLA-G1 cell-surface molecule was suggested to be a mechanism by which tumors render lymphocytes anergic [28]. In contrast, it can be envisioned that the transfer of active Ras–a G-protein known to reverse T cell anergy [29] and promote the acquisition of proinflammatory effector functions–from tumor cells to lymphocytes may serve as a ‘stimulatory’ signal for the immune system to eliminate nascent tumors expressing oncogenic forms of Ras. This hypothesis must be addressed in future experiments aimed at examining the functions of Ras transfer in vivo. Advances in whole-animal imaging and development of new animal models should allow studying the extent by which the transfer of oncogenic Ras and possibly other membrane-associated oncogenes influences the natural immunity to cancer.

## Materials and Methods

### Human subjects

This study was approved by the Institutional Ethics Committee at the Chaim Sheba Medical Center. All peripheral blood samples were obtained from healthy subjects.

### Antibodies and reagents

Red fluorescent-conjugated mouse mAbs directed against CD3, CD4, CD45, and CD56 were from BD Biosciences. Purified anti-pERK mAb was from Sigma, anti-pan Ras mAb from Calbiochem, anti-HA from Boehringer Mannheim, and both Alexa Fluor 647-conjugated secondary Abs and carboxyfluorescein diacetate succinimidyl ester (CFSE) were from Molecular Probes (Invitrogen).

### Plasmids and transfection

The expression vectors containing cDNA encoding GFP, GFP-H-Ras, GFP-H-RasG12V, and GFP-tH were previously described [9] B721 cells were transfected and selected to stably express the indicated vector as described elsewhere [30].

### Isolation of NK and T cells

Peripheral blood lymphocytes (PBLs) were isolated by density-gradient centrifugation on Histopaque 1077 (Sigma), as previously described [31]. Primary CD56+ NK cells were isolated from the PBLs by the use of anti-CD56 microbeads and the MACS® cell separation system (Miltenyi Biotec, Germany). To obtain a population enriched with CD3+ T cells, CD56^−^ PBLs were cultured for 16 h in plastic flasks and the non-adherent cells (∼85% CD3+ T cells) were collected.

### NK and T cell cultures

Cells were cultured in RPMI-1640 medium supplemented with 10% fetal bovine serum (FBS), 2 mM L-glutamine, 100 U/ml penicillin, and 100 µg/ml streptomycin, all from Gibco (Carlsbad, CA), and maintained at 37°C in a humidified 5% CO_2_ incubator. The CD56+ NK cells used for the H-Ras transfer experiments were plated beforehand into 24-well plates (Costar®) at 2×10^6^ cells/well and cultured for 24–48 h in medium supplemented with 100 IU of recombinant human interleukin (rhIL)-2 (Boehringer, Mannheim). To examine the effect of stimulation on the rate of H-Ras acquisition by T cells, the CD3+-enriched T cell cultures were activated by plate-bound anti-CD3 mAbs (2 µg/ml) and soluble anti-CD28 mAbs (1 µg/ml; BD Biosciences), and cultured for additional 3 days in a medium supplemented with 100 IU of rhIL-2.

### Cell lines and transfection

The human cell lines Jurkat, KG1, and K562 were cultured in RPMI-1640+10% FBS and maintained as above. The human B lymphoblastoid cell line 721.221 (B721) was obtained from the American Type Culture Collection. Transfection of B721 cells with cDNA expression vectors by electroporation and the generation of stable transfectants by G418 selection were done as previously described [30]. The human NK92 cell line (kindly provided by Ofer Mandelboim) and the CD4+HVS T cells (generated as previously described) [32] were cultured in RPMI-1640 medium+10% FBS supplemented with 100 IU of rhIL-2. COS-7 and HEK-293 cells were cultivated and transfected as described [9].

### Co-cultures and analysis of intercellular transfer

To examine the intercellular transfer of GFP-tagged molecules, the various B721-cell transfectants were distributed into U-bottom 96-well plates (3×10^4 ^cells/well in 100 µl) to which were added NK cells or T cells (6×10^4^ cells/well in 100 µl) to obtain an effector to target ratio of 2∶1. The culture plates were centrifuged for 2 min at 1,000 rpm to promote cell-conjugate formation and then co-cultured for 2–4 h at 37°C. For FACS-based analysis of GFP-H-RasG12V transfer, the collected cells were resuspended vigorously in 5 mM EDTA/PBS and kept on ice for 30 min to allow cell conjugates to dissociate. Immunofluorescence staining with anti-CD56, anti-CD3, or anti-CD45-allophycocyanin mAbs, as appropriate, was performed for 30 min at 4°C. After labeling the cells were washed and again resuspended in 5 mM PBS/EDTA. Data collected from ∼10 000 single-cell events were then analyzed by multiparametric FACS. Primary T cells or NK cells were distinguished from target cells by their smaller size (as defined by their FSC/SSC) and red fluorescence. In some experiments the effector cells were pretreated for 30 min with latrunculin B (1 µM) and co-cultured for 3 h with B721 cells in the presence of the drug or co-cultured with B721 cells in serum-free RPMI-1640 medium+5 mM EDTA.

### Cell-conjugate formation

For conjugation assays, NK cells or T cells were pre-labeled with anti-CD45-allophycocyanin before being co-cultured with B721 at a ratio of 1∶1, and 45 min later the cells were fixed with 2% paraformaldehyde. Flow cytometry identified effector-B721 conjugates by their increased forward scatter characteristics (FSC) and by their being positive for both GFP and CD45. The percentage of NK cells in a conjugate was calculated as follows: (effector cells in conjugate)/(total number of conjugated and single effector cells) × 100%.

### Microscopy

NK or T cells (CD45^high^) were co-cultured with B721 (CD45^negative^) cells for 2–3 h at 37°C. For fluorescence staining, the confocal (Zeiss LSM510) and epifluorescence microscopy (Olympus IX81) the cell-conjugates were fixed in 2% paraformaldehyde, cytospun onto slides, and stained with anti-CD45 phycoerythrin-Cy5 mAbs.

### Transwell assay

Effector T cells and NK cells were prevented from directly contacting the B721 by a Transwell 0.4-µm-pore membrane (Costar®). Briefly, 5×105 effector cells (in 0.3 ml of medium) were either added to B721−GFP-H-RasG12V cells (in 0.5 ml of medium) in the lower compartment (done in 24-well plates) or placed in the upper chamber separated from targets. The cells were incubated for 3 h at 37°C, and cells from the lower chamber were then collected in 5 mM EDTA/PBS and analyzed for GFP acquisition by the NK cells or the T cells as described above.

### FACS analysis and cell sorting

Cell samples were analyzed on a FACSCalibur™ using Cellquest™ software or on a FACSAria™ using FACSDiva™ software (all from BD Biosciences). Most of the FACS data analysis was done using FlowJo 7.2.1 software (Tree Star). All cell-sorting experiments were performed on FACSAria™. To obtain a single-cell suspension, cells were pre-treated (as described above) so as to dissociate cell-conjugates and were sorted at 4°C. To sort out only viable T- or NK-singlet cells we employed a stringent multiparametric gating strategy. Viable lymphocytes were identified by their distinct FSC and SSC (including pulse width, height and area), propidium iodide exclusion, and expression of a distinct NK- or T-cell marker as indicated (e.g. CD56, CD3, or CD45).

### Intracellular staining for flow cytometry

Cells were fixed in 2% paraformaldehyde for 10 minutes and permeabilized with methanol as previously described [29], [33]. Thereafter the cells were stained with the indicated primary mAbs for 20 min at room temperature, washed and stained with the secondary goat anti-mouse-Alexa Fluor 647 antibodies. The cells were then analyzed by FACS. Approximately 20,000 events were collected for each sample.

### Mixed lymphocyte reaction and proliferation assay

Freshly purified CD56− PBLs were labeled with CFSE as previously described [34]. PBL samples were then placed in U-bottom 96-well plates (1×10^5^ cells/well in 100 µl of medium) to which irradiated (5,000 rad) B721−GFP or B721−GFP-H-RasG12V had been added (3×10^4^ cells/well in 100 µl of medium), and cultured for 7 more days. The collected cells were stained with anti-CD3-allophycocyanin and the T cells were analyzed for CFSE dilution by FACS.

### Cytometric bead array (CBA)-based cytokine detection

Purified CD56− PBLs or CD56+ NK cells were placed in U-bottom 96-well plates (1×10^5 ^cells/well in 100 µl of medium) to which were added B721−GFP or B721−GFP-H-RasG12V cells (5×10^4^ cells/well in 100 µl of medium). Supernatants were collected 24 h later, and levels of IL-2, IL-4, IL-5, IL-10, interferon (IFN)-γ, and tumor necrosis factor-α were assayed, as previously described, using the human Th1/Th2 CBA kit (BD Biosciences). Data were collected on FACSCalibur™ and analyzed using the BD™ CBA software, according to the manufacturer's instructions.

### Fluorolysis assay

This assay, adopted from Kienzle et al [35] measures by flow cytometry the lytic activity of effector cells by counting the number of viable GFP^+^ target cells (as determined by propidium iodide exclusion) that were left after incubation with the NK effectors. Briefly, the various B721-cell transfectants were distributed into U-bottom 96-well plates (3×10^4 ^cells/well in 100 µl) to which NK cells were added (in 100 µl) to obtain different effector to target ratios. The culture plates were centrifuged for 2 min at 1,000 rpm and then co-cultured for 90 min at 37°C. The percentage of cell lysis was calculated by the following formula: 100−[100 (number of GFP^+ ^PI^−^ cells of sample)/(number of GFP^+^ PI^−^ cells of B721 cells only)]. For standardization of counted events, an equal number of the PE-labeled calibration beads were accumulated for each FACS run.

### Statistical analysis

*P* values were calculated by either the Student's t test or the non-parametric Wilcoxon signed rank test as appropriate. *P*<0.05 was considered significant.

## Supporting Information

Supporting Text S1 (0.09 MB DOC)

Figure S1 (0.35 MB TIF)

Figure S2 (0.45 MB TIF)

Figure S3 (1.27 MB TIF)

Figure S4 (0.44 MB TIF)

Figure S5 (0.89 MB TIF)

Figure S6 (0.59 MB TIF)

Figure S7 (0.76 MB TIF)

Figure S8 (0.33 MB TIF)

Figure S9 (1.08 MB TIF)
