# Supplementary material for: Intercellular Transfer of Oncogenic H-Ras at the Immunological Synapse
Source: PLoS One. 2007 Nov 21;2(11):e1204. doi: 10.1371/journal.pone.0001204 (PMC2065899; doi:10.1371/journal.pone.0001204)
Supplement: Supporting Text S1 — (0.09 MB DOC) [file pone.0001204.s001.doc]

Supporting Text S1

Intercellular Transfer of Oncogenic H-Ras at the Immunological Synapse

Oded Rechavi[†], Itamar Goldstein[†]*, Helly Vernitsky, Barak Rotblat & Yoel Kloog*

[†]These authors contributed equally to this work.

*Correspondence should be addressed to Y.K. (kloog@post.tau.ac.il); or I.G. (Itamar.Goldstein@sheba.health.gov.il).

This file includes:

Figure legends S1-S9

Fig. S1 Kinetics of GFP-H-RasG12V transfer. Freshly isolated CD56+ NK cells from healthy subjects were co-cultured with B721−GFP cells or B721−GFP-G12V cells for the indicated periods. The collected cells were treated as described in methods to disrupt cell conjugates, stained with allophycocyanin-conjugated anti-CD56 mAbs, and analyzed by FACS. Results shown are of a typical experiment out of three each performed in duplicates. Data were collected from ~10 000 single cell events and are presented as percentage of NK cells that acquired GFP or GFP-H-Ras-G12V.

Fig. S2. The transferred GFP-H-RasG12V is localized inside the acquiring NK cells. **(a-d)** CD56+ NK cells were co-cultured with B721−GFP cells (left panel) or B721−GFP-H-Ras-G12V (right panel) for 2 hours. Thereafter the collected cells were fixed / permeabilized, and stained with anti-pan-Ras mAb, followed by Alexa Fluor 647 goat anti-mouse antibodies. The cells were then analyzed on a FACSCalibur™, and the zebra plots (a mix of contour and density plot) depict either the B721-gate (**a-b)** or the NK-gate **(c-d)**. Cells in the upper right quadrant of figure **d** represent NK cells positive for both GFP and intracellular Ras. **(e-f)** CD56+ NK cells were co-cultured with B721−GFP cells **(e)** or B721−GFP-H-Ras-G12V **(f)**, collected and stained for cell surface bound Ras with anti-pan-Ras mAb, followed by Alexa Fluor 647 goat anti-mouse antibodies. The results shown are of a typical experiment (one of more than three performed).

Fig. S3. The human NK92 and Jurkat T-cell lines also acquire GFP-H-RasG12V from B721 transfectants. NK92 cells (upper panel) or Jurkat cells (lower panel) were co-cultured with B721−GFP cells (left panel) or B721−GFP-H-RasG12V (right panel). The collected cells were pretreated to disrupt conjugates as described in Methods, stained with anti-CD45-allophycocyanin, and analyzed on a FACSCalibur™. Note that unlike B cells the B721 cell line is CD45 negative. Values in each quadrant represent the numbers of events expressed as percentages of the total number. In each case the results shown are of a typical experiment (one of more than three performed).

Fig. S4. The Ability to acquire GFP-H-RasG12V from conjugated partners is a lymphocytic characteristic. Different cell types (effectors) as indicated were co-cultured with B721−GFP-H-RasG12V for 3 h and the percentages of their GFP+ effectors were then determined by FACS analysis. Bar graphs represent the extent of transfer in the various cell types relatively to the transfer recorded in T cells. Experiments were performed in duplicate and bars depict the mean results. A typical experiment of > 3 experiments performed is shown.

Fig. S5. Lymphocytes acquire GFP-H-RasG12V from non-classical antigen-presenting cells. Activated CD3+ T cells were co-cultured for 3 h with COS or HEK293 cells transfected to express GFP or GFP-H-RasG12V. The cells were treated as described in Methods, stained with anti-CD3- allophycocyanin mAbs, and analyzed on a FACSCalibur™. Bar graphs represent the numbers of GFP+ T cells expressed as

percentages of the total T-cell number. The results shown are of a typical experiment (one of more than five performed).

Fig. S6. Intercellular transfer of oncogenic H-Ras is independent of the GFP fusion protein. NK cells were co-cultured for 2 h with native HEK293 or HEK293 transfected with HA-H-RasG12V. The cells were collected, fixed / permeabilized, and stained with anti-HA mAbs followed by secondary Alexa Fluor 647 goat anti-mouse antibodies. The cells were then analyzed on a FACSCalibur™. Overlay histograms depict total HA levels in either NK cells **(a)** or HEK293 **(b)**. The results shown are of a typical experiment (one of the 3 performed).

Fig. S7. Inhibitors of Ras transfer also disrupt cell conjugate formation. PBLs were co-cultured for 45 min with B721−GFP-H-RasG12V at a ratio of 1:1, and the percentage of cell conjugates was then determined by FACS analysis (see also Fig. 4b). The co-cultures were treated as indicated or left untreated. Experiments were performed in duplicates (data from ~10,000 single cell events) and bars represent mean ± SD. Shown is a typical experiment out of more than 3 experiments performed.

Fig. S8. Lymphocytes acquire GFP-H-Ras wt and the GFP-tH from target cells. Activated CD3+ T cells were co-cultured for 3 h with HEK293 cells transfected to express GFP-H-Ras(wt), GFP-H-RasG12V, GFP-tH, or GFP. The cells were treated as described in Methods, stained with allophycocyanin-tagged anti-CD3 mAbs, and

analyzed on a FACSCalibur™. Bar graphs represent the numbers of GFP+ T cells expressed as percentages of the total T-cell number. The results shown are of a typical experiment (one of more than three performed).

Fig. S9 Trogocytosis and Ras acquisition are linked. Freshly isolated CD56+ NK cells were co-cultured with B721-GFP cells or B721-GFP-H-RasG12V cells for 2 hours. The collected cells were treated as described in methods to disrupt cell conjugates, stained with allophycocyanin-conjugated anti-CD86 mAbs, and analyzed by FACS. **(a)** Left dot plot depicts co-localization of GFP-H-RasG12V and CD86 in NK cells. Right plot shows the analysis of the raw data shown in (a) which were exported into Excel spreadsheet and analyzed using the MedCalc V6.14 software. Results shown are of a typical experiment out of three performed (data was collected from ~10 000 single cell events). **(b)** The overlay histograms depict CD86 levels in NK cells either cultured alone (red line), or co-cultured with the indicated B721 transfectants (green line).
