# Supplementary figures and images for: Intercellular Transfer of Oncogenic H-Ras at the Immunological Synapse
Source: PLoS One. 2007 Nov 21;2(11):e1204. doi: 10.1371/journal.pone.0001204 (PMC2065899; doi:10.1371/journal.pone.0001204)

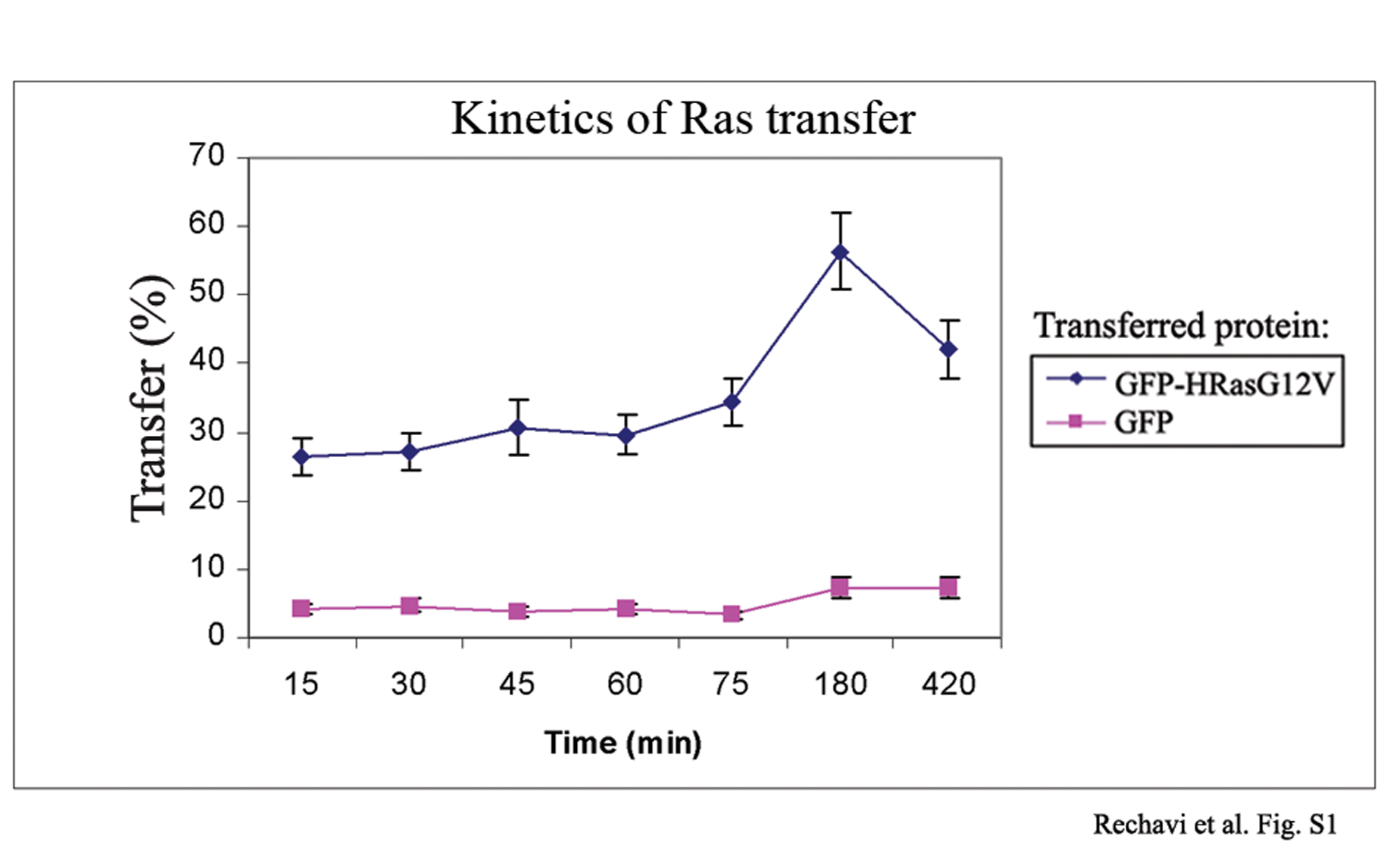

Supplement: Figure S1 — (0.35 MB TIF) [file pone.0001204.s002.tif]

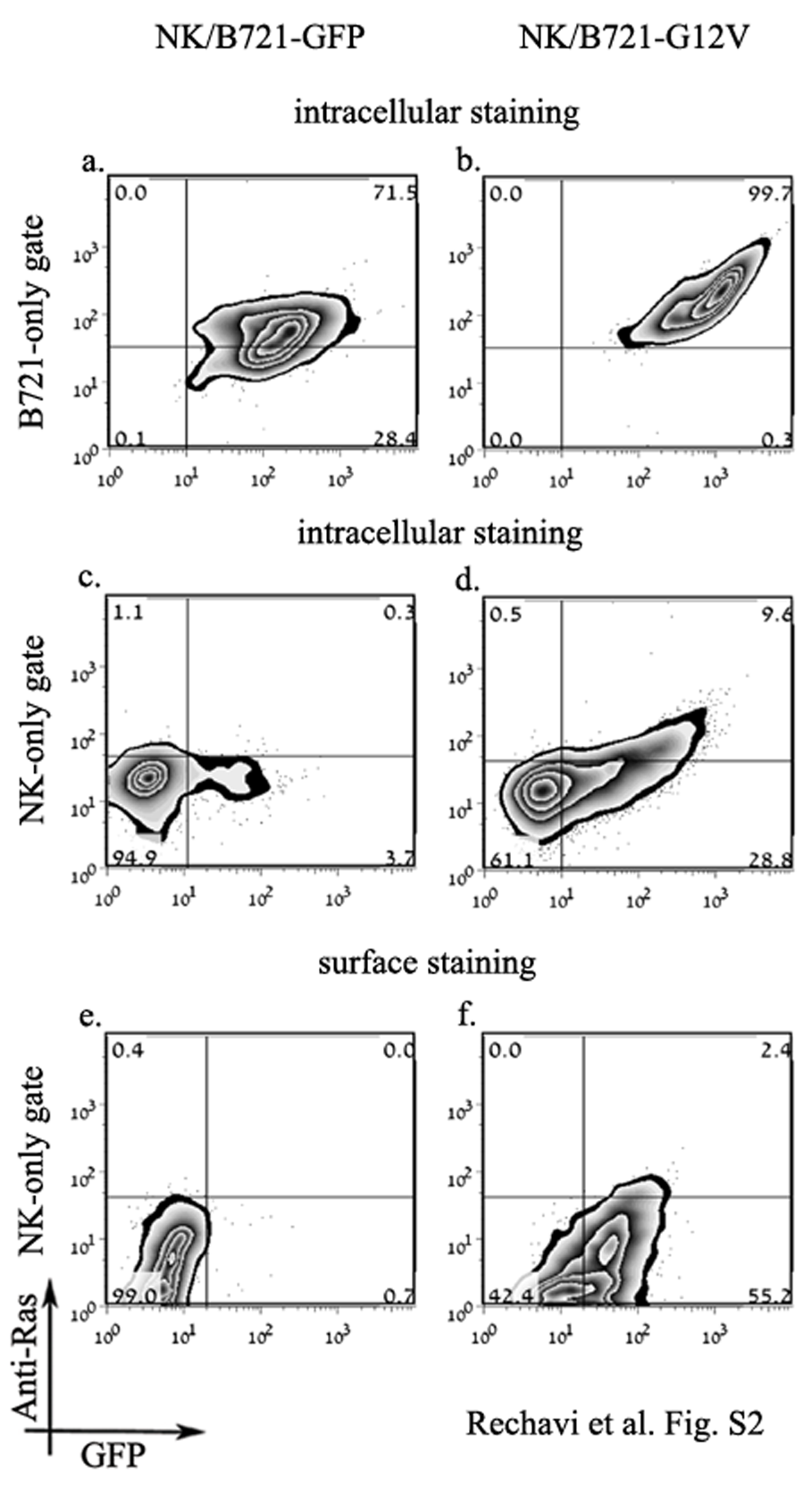

Supplement: Figure S2 — (0.45 MB TIF) [file pone.0001204.s003.tif]

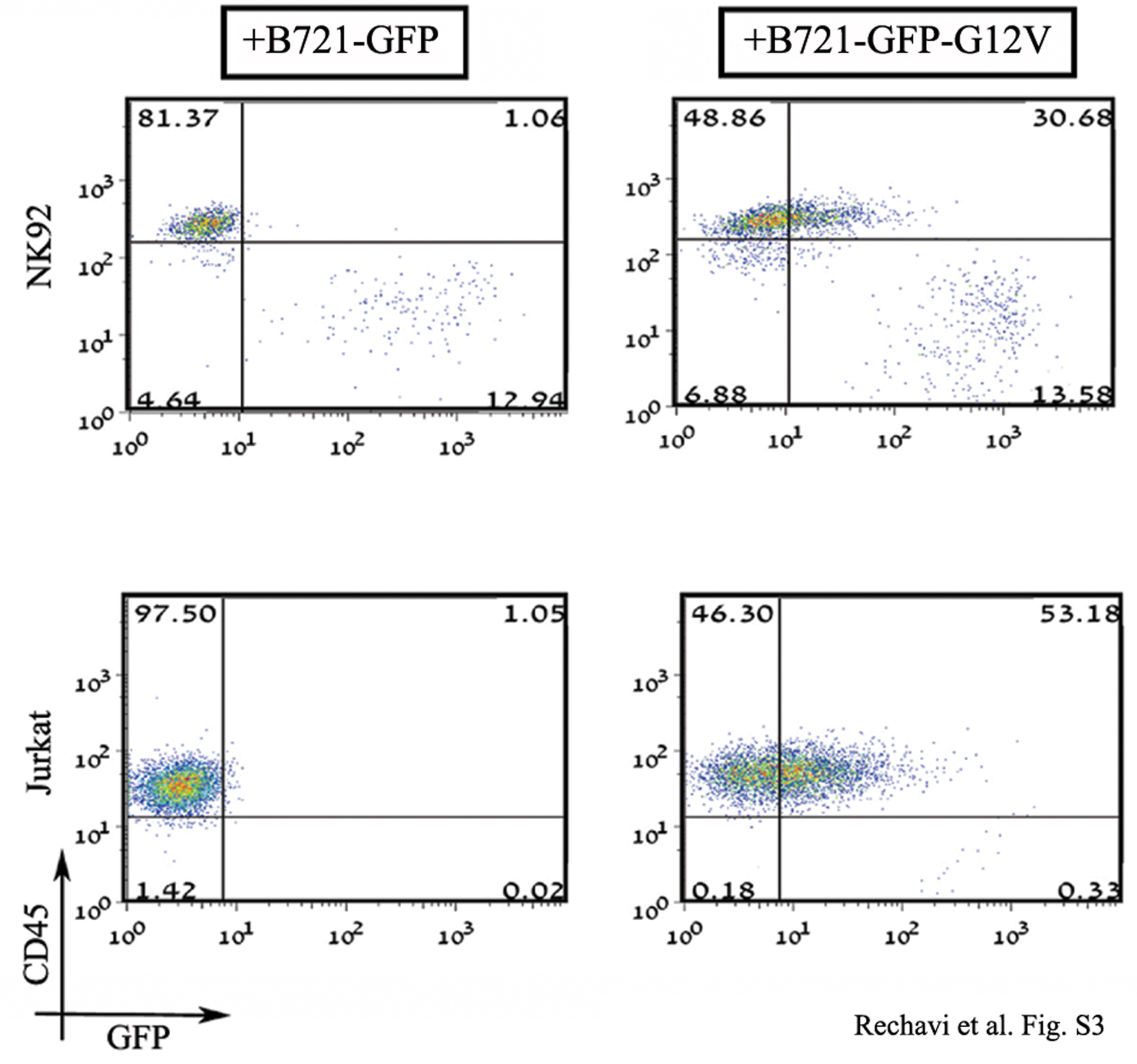

Supplement: Figure S3 — (1.27 MB TIF) [file pone.0001204.s004.tif]

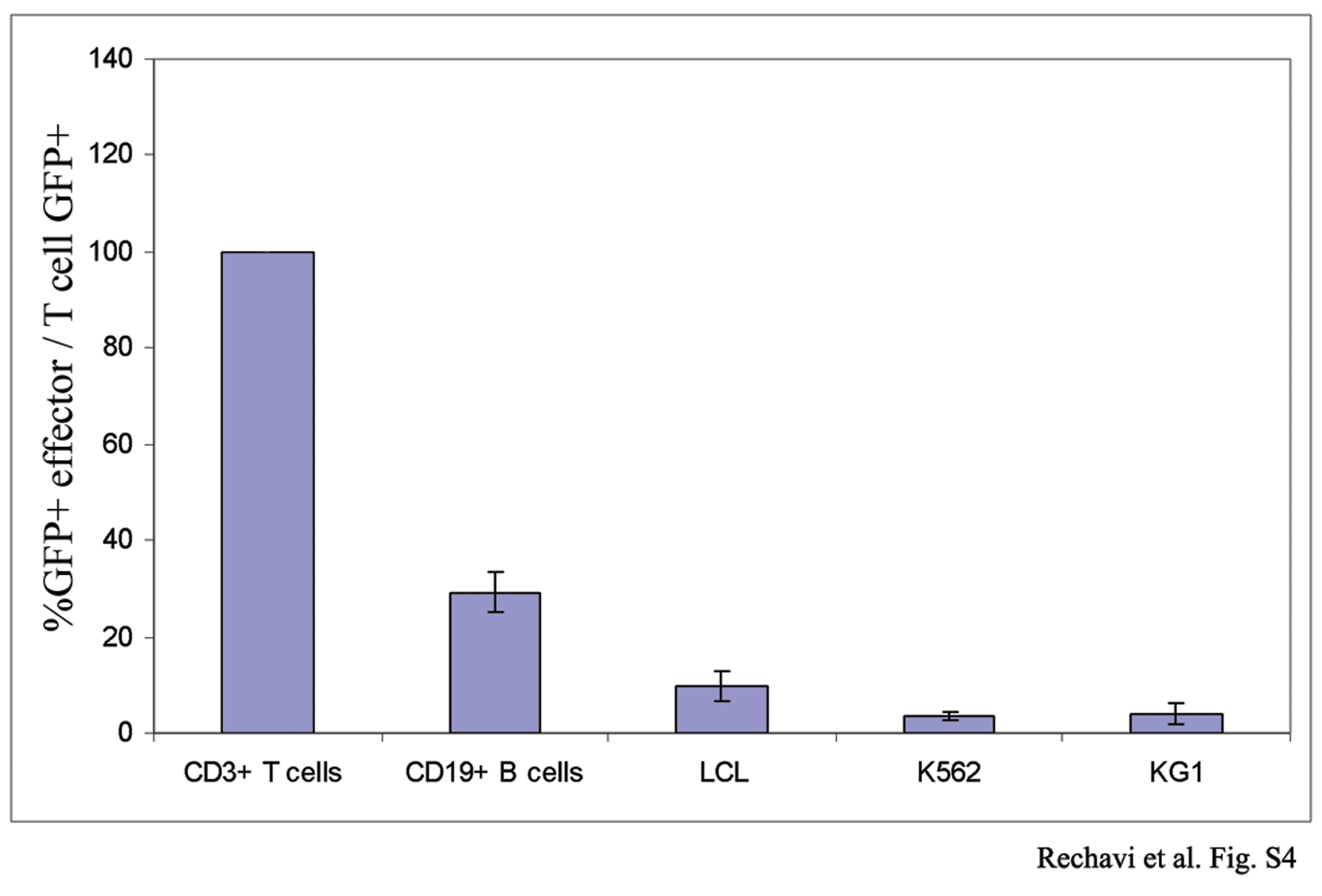

Supplement: Figure S4 — (0.44 MB TIF) [file pone.0001204.s005.tif]

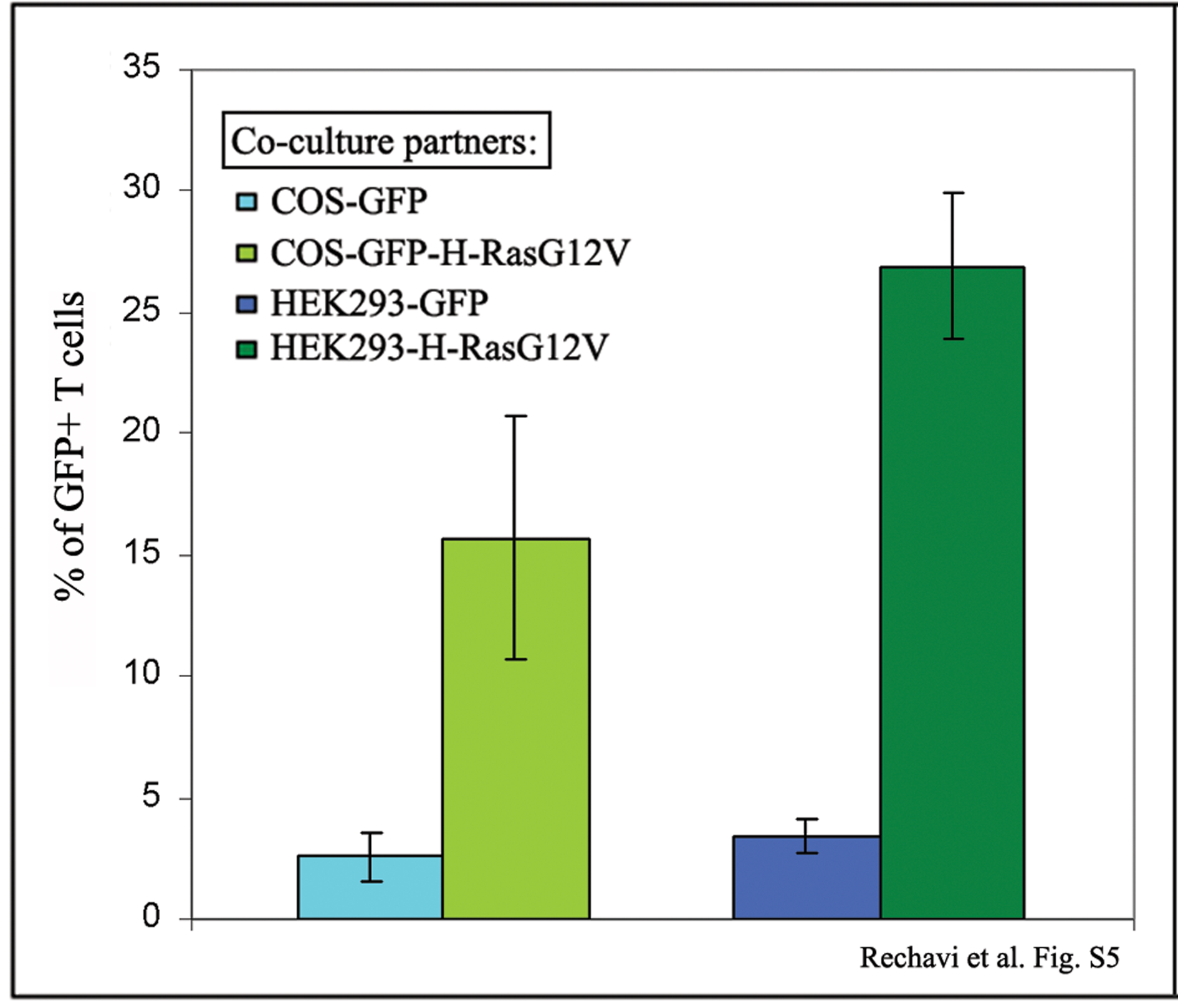

Supplement: Figure S5 — (0.89 MB TIF) [file pone.0001204.s006.tif]

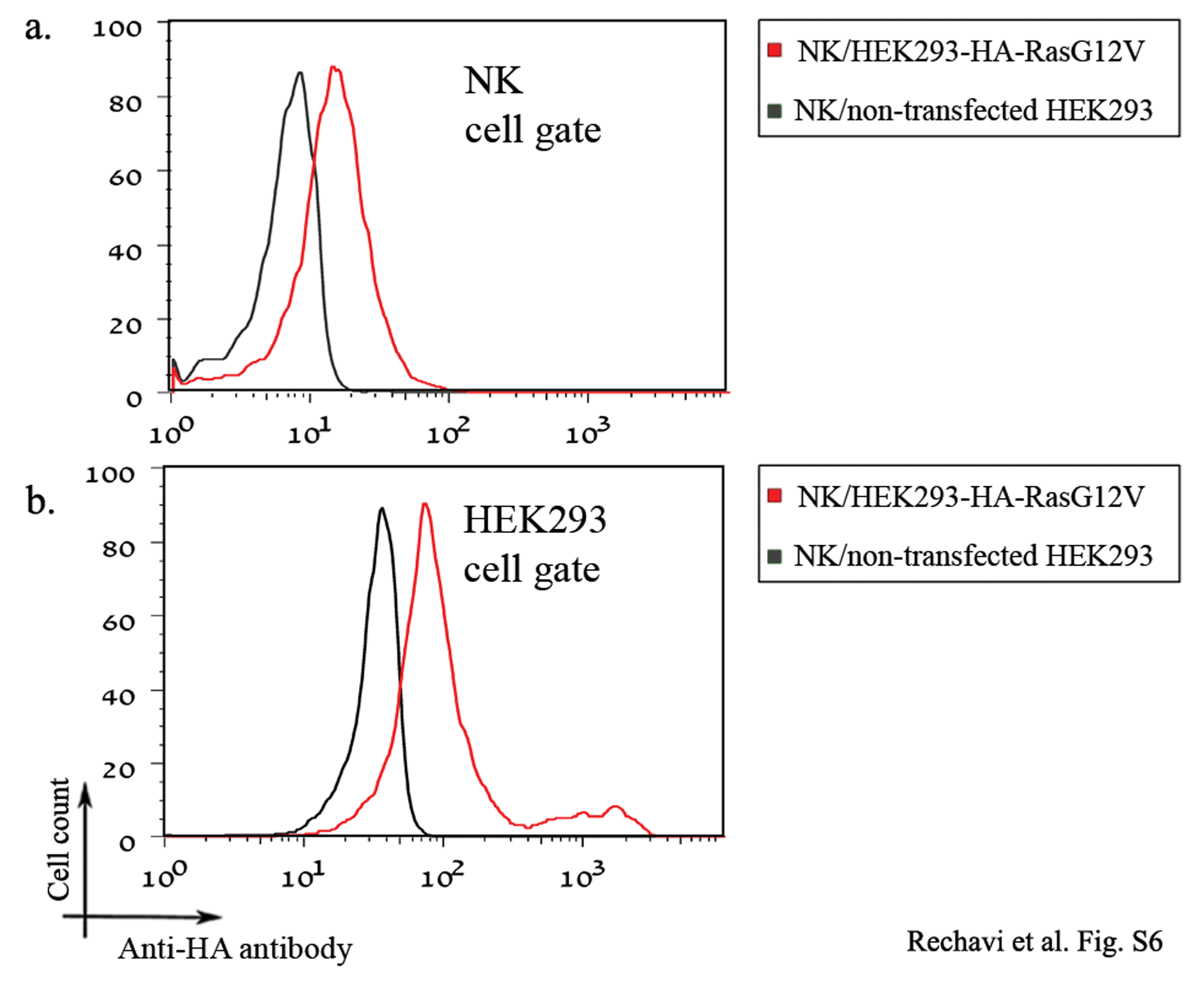

Supplement: Figure S6 — (0.59 MB TIF) [file pone.0001204.s007.tif]

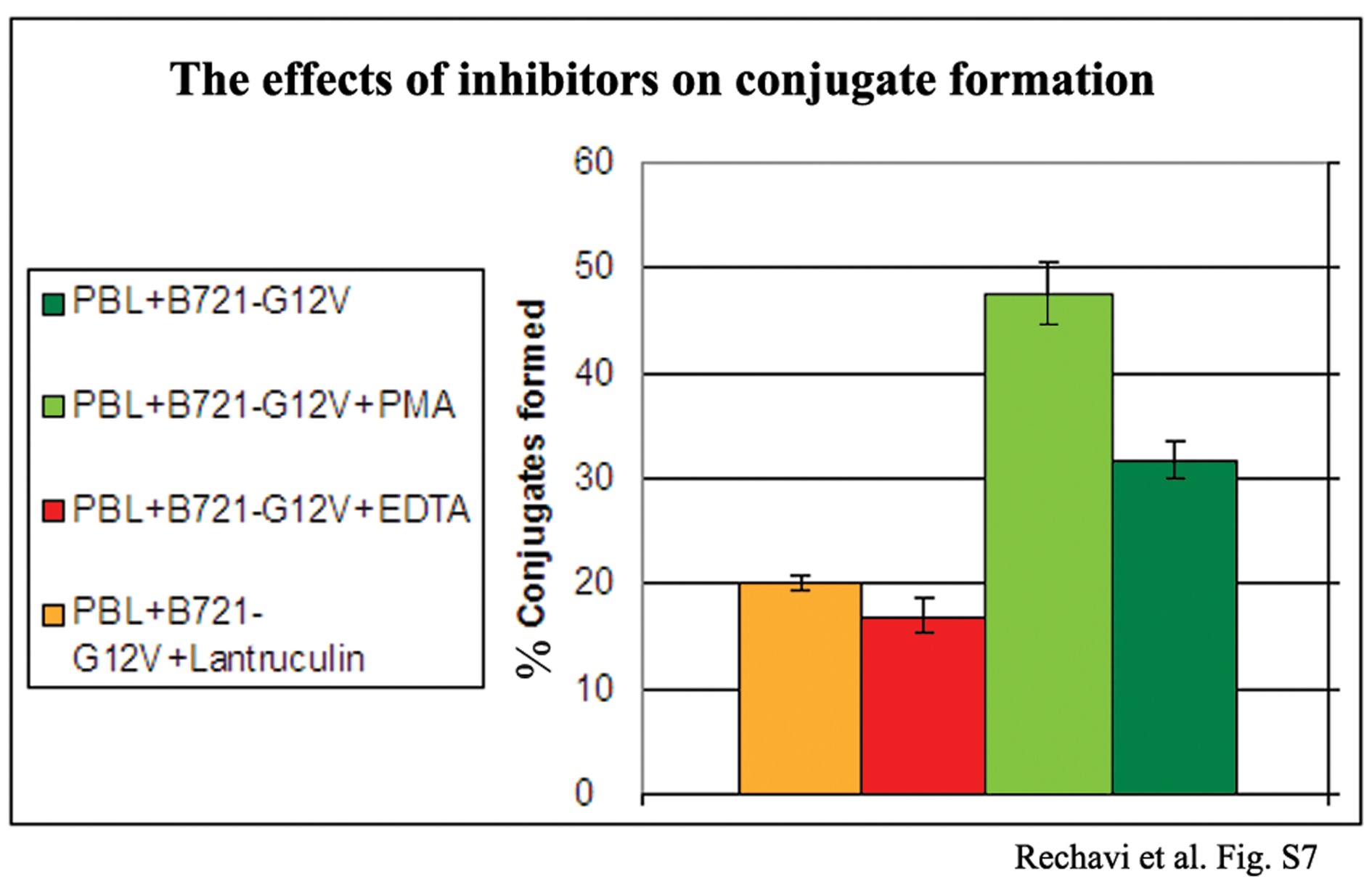

Supplement: Figure S7 — (0.76 MB TIF) [file pone.0001204.s008.tif]

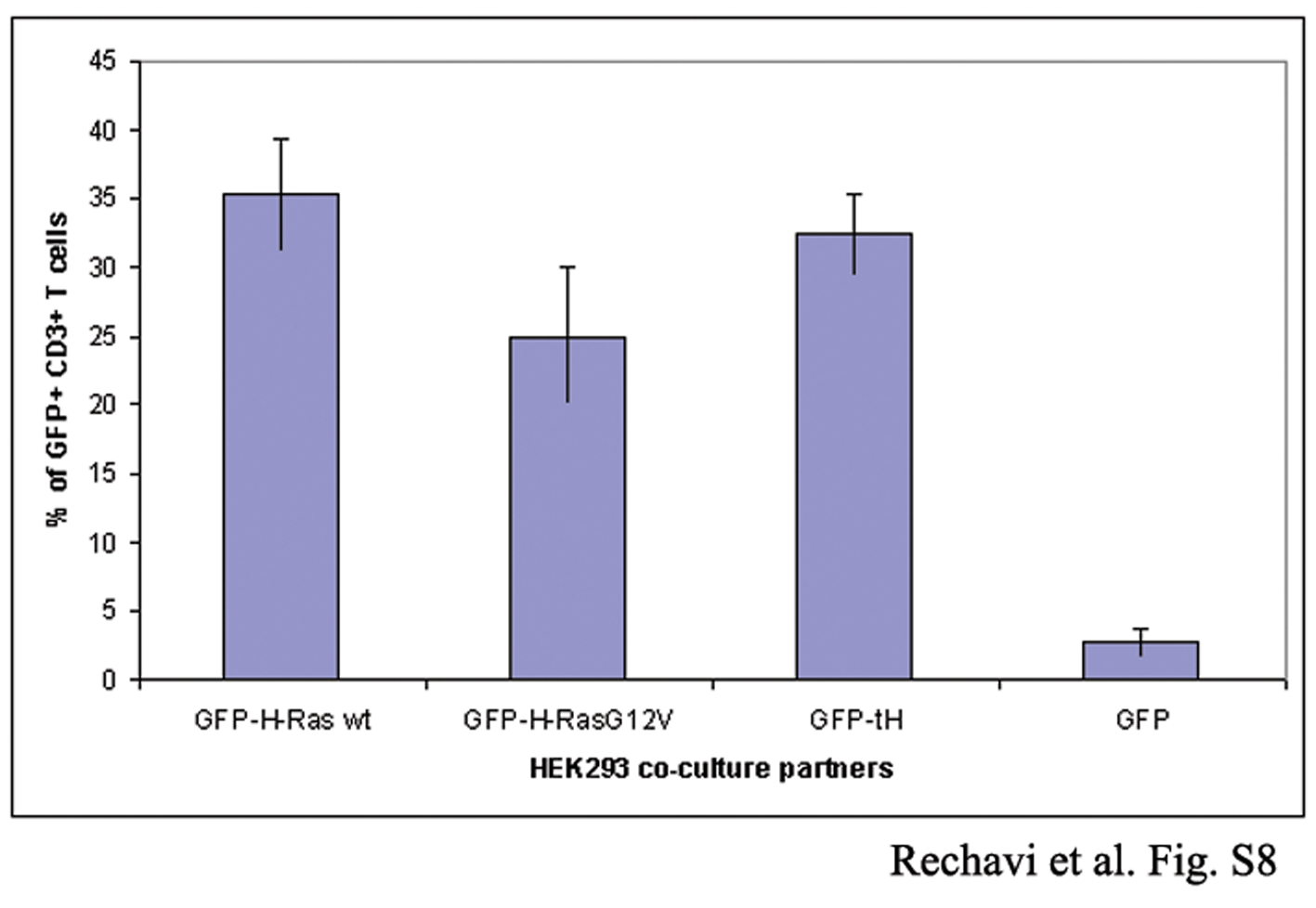

Supplement: Figure S8 — (0.33 MB TIF) [file pone.0001204.s009.tif]

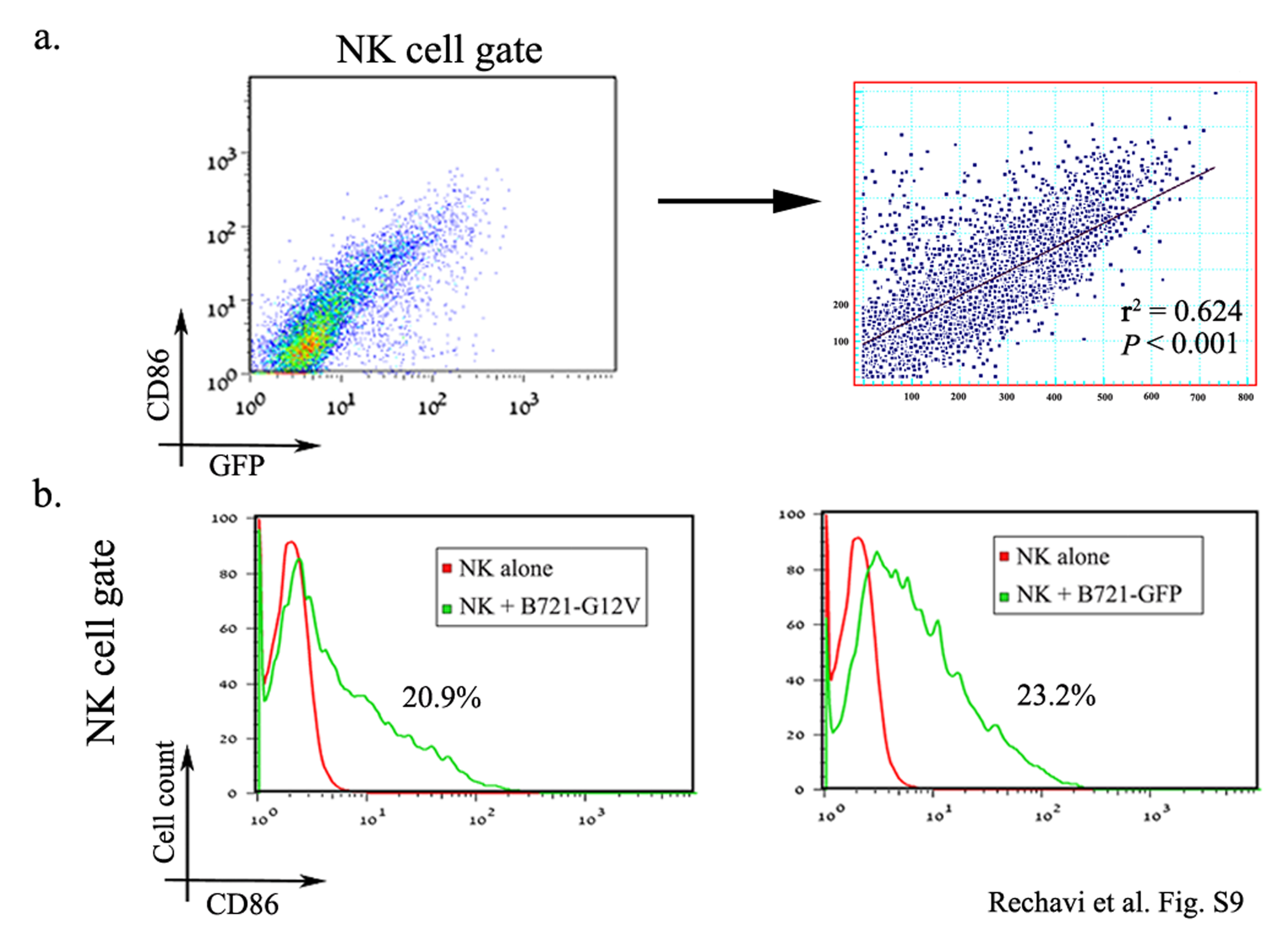

Supplement: Figure S9 — (1.08 MB TIF) [file pone.0001204.s010.tif]
